# Evolution and Transmission of Respiratory Syncytial Group A (RSV-A) Viruses in Guangdong, China 2008–2015

**DOI:** 10.3389/fmicb.2016.01263

**Published:** 2016-08-15

**Authors:** Lirong Zou, Lina Yi, Jie Wu, Yingchao Song, Guofeng Huang, Xin Zhang, Lijun Liang, Hanzhong Ni, Oliver G. Pybus, Changwen Ke, Jing Lu

**Affiliations:** ^1^Guangdong Provincial Center for Disease Control and PreventionGuangzhou, China; ^2^Guangdong Provincial Institute of Public Health, Guangdong Provincial Center for Disease Control and PreventionGuangzhou, China; ^3^Department of Zoology, University of OxfordOxford, UK

**Keywords:** respiratory syncytial virus, phylogenetic, phylogeographic, evolution, transmission

## Abstract

Respiratory syncytial viruses (RSVs) including subgroups A (RSV-A) and B (RSV-B) are an important cause of acute respiratory tract infections worldwide. RSV-A include major epidemic strains. Fundamental questions concerning the evolution, persistence and transmission of RSV-A are critical for disease control and prevention, yet remain unanswered. In this study, we generated 64 complete *G* gene sequences of RSV-A strains collected between 2008 and 2015 in Guangdong, China. Phylogenetic analysis was undertaken by incorporating 572 publicly available RSV-A sequences. Current data indicate that genotypes GA1, GA4, and GA5 are endemic with limited epidemic activity. In contrast, the GA2 genotype which likely originated in 1980 has spread rapidly and caused epidemics worldwide. By analyzing GA2 genotype sequences across epidemic seasons within Guangdong, we find that RSV-A epidemics in Guangdong are caused by a combination of virus importation and local persistence, although the magnitude of the latter is likely overestimated due to infrequent sampling in other regions. Our results provide new insights into RSV-A evolution and transmission at global and local scales and highlights the rapid and wide spread of genotype GA2 compared to other genotypes. In order to control RSV transmission and outbreak, both local persistence and external introduction should be taken into account when designing optimal strategies.

## Introduction

Human respiratory syncytial virus (RSV) is recognized as an important cause of acute respiratory tract infections (ARI), especially in children under 5 years old (Storey, 2010). The clinical manifestations of RSV infection range from mild symptoms in the upper respiratory tract to severe disease such as bronchiolitis and pneumonia (Welliver, 2003). RSV infection induces only partially protective immune responses that do not confer long-lasting protection (Gonzalez et al., 2012), therefore repeated infections of RSV are common (Henderson et al., 1979; Glezen et al., 1986). It has been estimated that RSV infects 70% of children during their first year of life and nearly all children older than two (Gonzalez et al., 2012). RSV infections cause an estimated 3.4 million children’s hospital admissions annually, leading to a huge medical burden (Nair et al., 2010).

Respiratory syncytial viruse strains are classified into two major subgroups, RSV-A and RSV-B, according to their antigenic and genetic variability (Mufson et al., 1985). The two subgroups are further classified into different genotypes according to the genetic divergence of the viral *G* gene (Botosso et al., 2009). RSV-A include major epidemic strains (Scott et al., 2006). Based on *G* gene phylogenies, RSV-A can be classified into at least seven genotypes (GA1-7) (Trento et al., 2015). Co-circulation of different RSV-A genotypes in a population was previously considered as a reason for repeated infection and annual viral outbreaks (Garcia et al., 1994). However, recent surveillance has suggested that a single genotype of RSV-A, GA2, has spread internationally and become predominant in successive epidemic seasons (Eshaghi et al., 2012; Houspie et al., 2013; Agoti et al., 2014; Liu et al., 2014; Pierangeli et al., 2014; Duvvuri et al., 2015).

The prevention and control of RSV relies on our understanding of the virus’ evolution and dissemination. Questions such as how RSV epidemics occur persist and reappear at global and local scales are important for designing optimal surveillance and prevention strategies, but remain largely unanswered (Hirano et al., 2014; Bose et al., 2015; Kimura et al., 2016). RSV is also proved as one of major etiologies of ARIs in mainland China (Liu et al., 2014; Dong et al., 2016; Fan et al., 2016). However, the dynamics of RSV infections in China are not clearly illustrated due to a lack of continuous surveillance on RSV epidemics. In this study, we collected RSV-A strains between 2008 and 2015 in Guangdong, China. These were sequenced and combined with other publicly available RSV-A sequences. We undertook phylogenetic, spatial and molecular clock analyses to investigate the molecular epidemiology of RSV-A at both global and local scales.

## Materials and Methods

### Ethics Statement

In this study, all analyses were performed anonymously and did not involve human experimentation. This study was approved by the Ethics Review Committee of the Guangdong Center for Disease Control and Prevention. Respiratory samples were collected from patients in accordance with the guidelines of the Ministry of Health, P. R. of China, for public health purposes. Written consent was prepared and signed by all of patients or their guardian(s) when samples were collected.

### Clinical Samples

Respiratory syncytial viruse surveillance was performed in four sentinel hospitals (Sun Yat-Sen Memorial Hospital, Guangdong No.2 Provincial People’s Hospital,Guangzhou Women and Children’s Medical Center and The Second Affiliated Hospital of Guangzhou Medical University) in Guangzhou, the capital city of Guangdong Province, from January 2008 to December 2015. Patients suspected of having ARIs from both inpatient and outpatient were enrolled according to these criteria: acute fever (*T* ≥ 38°C), and/or abnormal leukocyte count, with any one respiratory symptom (such as sore throat, cough, expectoration, and dyspnoea/tachypnoea). Nasopharyngeal swabs (NPSs) were collected within 24 h after admission. The age and sex distributions of patient are shown in Supplementary Table S2.

### Viral Test and RSV Sequencing

Nasopharyngeal swabs were kept and transported in viral transport medium and stored at -70°C prior to analysis. Total viral nucleic acids (DNA and RNA) were extracted using QIAamp MiniElute Virus Spin kits (Qiagen, Valencia, CA, USA) according to the manufacturer’s instructions. For each specimen, RSV infection was detected by using qRT-PCR with a QIAGEN OneStep RT-PCR Kit and RSV *F* gene specific primers and a probe, RSV-F: 5′-GCGTAACWACACCTKTAAGCACT-3′ and RSV-R: 5′-CTTTGCTGYCTWACTATYTGAACATTG-3′ RSV-Probe: FAM-ATCAATGATATGCCTATAACAAATGA-BHQ1. PCR was performed with the following thermal profile: reverse transcription at 45°C for 10 min, followed by 10 min at 95°C; and 40 cycles of 95°C 15 s and 55°C 60 s. RSV-positive confirmed samples were further screened for subgroup (A/B) by amplifying and sequencing the full length of *G* genes which were further used for phylogenetic analysis. Briefly, RNA was firstly reverse transcribed into cDNA using random hexamer primers and SuperScript II Reverse Transcriptase (Invitrogen, USA). Then the *G* gene was amplified by using nested PCR with Taq PCR Master Mix Kit (Qiagen). The primers used for RSV-A are as follow: RSVA-F1 (5′-TCAAGCAAATTCTGGCCTTA-3′) and RSVA-R1 (5′-CAACTGCAATTCTGTTTACAGCA-3′); RSVA-F2 (5′-CCTTTGAGCTACCAAGAGCTC-3′) and RSVA-R2 (5′-GAGTGTGACTGCAGCAAGGA-3′). PCR was performed with the following thermal profile: 94°C 3 min, 40 cycles of 94°C 30 s, 52°C 60 s, and 72°C 1 min 40 s and followed by final extension at 72°C 10 min. Around 1300 bps PCR products were sequenced using an ABI3730xl DNA Analyzer at IGE Biotech Co., Ltd. (Guangzhou, China). The RSV-A sequences generated in this study have been submitted to GenBank (accession numbers KX009654- KX009717). Some RSV-A sequences collected from one sentinel hospital (Guangzhou Women and Children’s Medical Center) between 2011 and 2013 were previously submitted and were included in analysis of virus local transmission (**Figure 3**).

### Sequence Alignment and Maximum-Likelihood Phylogenetic Analysis

RSV-A sequences generated in this study were combined with all publicly available RSV-A *G* gene sequences with known sampling date and known sampling location in GenBank^1^. Partial sequences covering different parts of the *G* gene were excluded and identical sequences collected in the same sampling location on the same date were removed to improve computation time. In total, 572 sequences that covered at least 95% of *G* gene were included in the phylogenetic analysis (Supplementary Table S3). Multiple sequence alignment was performed using ClustalW (Larkin et al., 2007) and alignments were minimally edited by hand using Aliview (Larsson, 2014). Recombination was checked by using the GARD tools (Kosakovsky Pond et al., 2006) available from the Datamonkey facility^2^, which did not yield any indications of recombination being present in our data sets. The best-fit nucleotide substitution model (GTR + G) were selected by using W-IQ-TREE with the Bayesian information criterion (Trifinopoulos et al., 2016). Temporal accumulation of genetic divergence was assessed from maximum likelihood mid-point rooted phylogenies using the linear regression approach implemented in TempEst (formerly Path-O-Gen) (Rambaut et al., 2016).

### Dated Phylogenetic Analysis

Bayesian Markov chain Monte Carlo (MCMC) phylogenetic inference was performed using BEAST, under a GTR + G substitution model (Shapiro et al., 2006) and a GMRF Bayesian skyride coalescent model (Minin et al., 2008). Preliminary analysis indicated high values for the coefficient of variation parameter of the molecular clock model, therefore an uncorrelated lognormal (UCLD) relaxed clock model was used in the final analysis to accommodate variation in substitution rates among branches (Drummond et al., 2006). Three independent MCMC runs of 1 × 10^8^ steps were computed and 10–20% burn-in was discarded from each, resulting in a total of 2.0 × 10^8^ total steps. Model parameters and trees were sampled every 10,000 MCMC steps. Convergence and behavior of MCMC chains was inspected using Tracer v1.6^3^ (Lemey et al., 2014). A subset of 500 trees was randomly drawn from the combined posterior distribution of trees and used as an empirical distribution for subsequent phylogeographic analysis (Lemey et al., 2014). Maximum clade credibility (MCC) phylogenetic trees were also estimated for representative Guangdong RSV-A sequences and closely related sequences by setting strong priors value on virus evolution rates.

### Phylogeography

We employed a Bayesian discrete phylogeographic approach to investigate viral spatial movement among four geographic regions (Africa, America, Asia, and Europe; **Figure 3**). Two RSV-A sequences from Australia were included in the Asia group. To ensure a realistic model of the direction of virus transmission, we used an asymmetric continuous-time Markov chain (CTMC) model (Edwards et al., 2011) to estimate ancestral locations and to estimate location posterior probabilities for each node in the time-scaled phylogenies.

## Results

### Epidemiology of RSV in Guangdong

**FIGURE 1:**
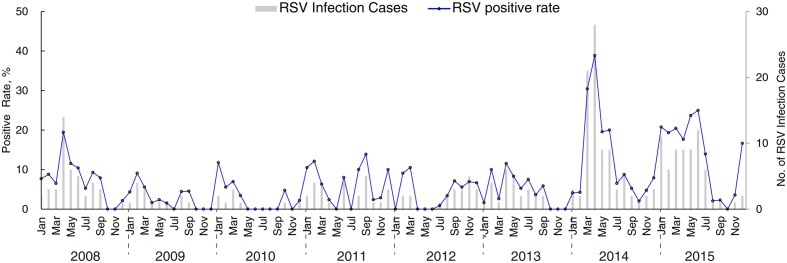
**Temporal distribution of RSV infections cases and RSV positive rates in acute respiratory tract infection samples collected in Guangzhou, Guangdong province, from 2008 to 2015**.

A total of 3843 ARI samples were collected from four surveillance hospitals in Guangzhou, the provincial capital of Guangdong, China, between 2008 and 2015. RSV was detected in 295 samples (7.68%, Supplementary Table S1). The seasonal distribution of RSV cases and the frequency of RSV positivity in ARI cases are shown in **Figure 1**. Although RSV infection peaks in each winter, from December to March, the seasonality of RSV infection in Guangdong is obscure, as a high relative risk of RSV infection is sometimes also observed in other seasons, e.g., in September 2011 and June 2015 (**Figure 1**). Notably, RSV epidemic activity was substantially increased in 2014 and 2015, with both more infection cases and higher positive rates in ARI samples in these years (**Figure 1**; Supplementary Table S1). RSV was detected in 14.1 and 13.8% of all tested samples collected in 2014 and 2015, which represents an almost threefold increase on the values for 2009–2013 (Supplementary Table S1). In total, 122 RSV positive samples were successfully sequenced in order to obtain full length *G* gene sequences; 64 samples were classified into the RSV-A subgroup through sequence alignment.

### Genetic Evolution of RSV-A Through History

**FIGURE 2:**
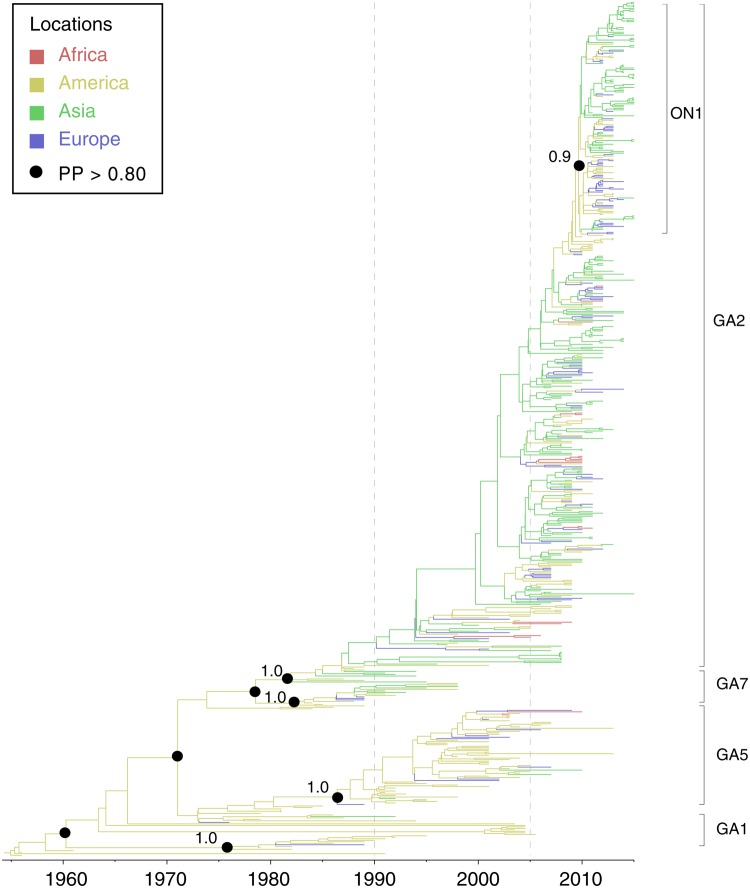
**Molecular clock phylogeny of global RSV-A *G* gene sequences**. The tree shown is a maximum clade credibility (MCC) phylogeny, Branch colors represent the most probable ancestral locations of each branch, inferred using a phylogeographic model (see Materials and Methods for details). Four major genotypes of RSV-A are denoted on the right. Black circles indicate posterior probabilities >0.80 at selected nodes. Location posterior probabilities are noted above specific branches. Transverse axis shows time line in units of years.

Spatial and temporal phylogenetic analyses were performed to describe the evolution of RSV-A. All other publicly available *G* gene sequences of RSV-A were combined with the new sequences generated in this study (see Materials and Methods). The evolutionary rate for RSV-A was estimated to be 2.3 × 10^-3^ substitutions/site/year (95% highest posterior density interval, HPD = 2.0–2.6 × 10^-3^ substitutions/site/year). As **Figure 2** shows, the RSV-A *G* gene phylogeny was classified into major RSV genotypes, as previously described (Peret et al., 1998). Genotype GA1 represents older strains, mainly sampled between 1980 and 1990. Strains near the root of GA1 genotype primarily circulated in the USA (**Figure 2**). In addition, the spread of GA1 viruses appears to be geographically limited as most sequences in this genotype were identified in America. Although an American origin for the GA1 genotype seems the most probable (posterior probability = 1.0), this might be also caused by sampling biases (**Figure 2**). The GA1 genotype was rarely detected in the last 10 years, indicating that this genotype may be extinct or cause only sporadic infections. In comparison, the GA5 genotype has been continuously detected in the USA from 1979 to 2013 (**Figure 2**). Occasional GA5 infections have been also reported in countries on other continents, including Netherlands (Tan et al., 2012), Spain (Trento et al., 2015), Viet Nam (Do et al., 2015), and South Africa (Pretorius et al., 2013). Despite this, more than 90% of GA5 genotype strains were identified in American countries (**Figure 2**).

Genotypes GA2 and GA7 are closely related in the phylogenetic tree. The common ancestor of these two genotypes descended from an ancestral lineage that can be date to around 1978 (1970–1975, 95% HPD). GA7 viruses circulated for an only short period (1984–1998) and were rarely detected after 2000. GA2 viruses share a common ancestor around 1980 (1978–1982, 95% HPD) and have become the most prevalent genotype in the last decade. In contrast to the “endemic” pattern observed for genotypes GA1 and GA5, GA2 spread to countries outside the Americas after its emergence. Strains from different locations including Uruguay, the USA, Netherland and Korea contribute to the “trunk lineage” of the GA2 genotype (sequences collected 1990–2005, **Figure 2**). The geographic spread of RSV-A GA2 was particularly pronounced after 2005 (**Figure 2**). After this, a significant change in RSV epidemiology was observed in different locations, with a shift from the circulation of multiple genotypes to prolonged circulation of predominant genotype GA2 (Botosso et al., 2009; Houspie et al., 2013). Most recently, the new variant of GA2, termed ON1, was identified in Ontario (Canada) and Panama in 2010 (Eshaghi et al., 2012). The ON1 genotype has spread widely in a short period of time, notably in 2011–2012, (**Figure 2**) and was reported as the dominant RSV-A strain in Europe in 2012–2013 (Pierangeli et al., 2014; Trento et al., 2015), in Africa in 2012 (Agoti et al., 2014) and in Asia in 2014 (Liu et al., 2014).

### RSV-A Infection in Guangdong 2008–2015

**FIGURE 3:**
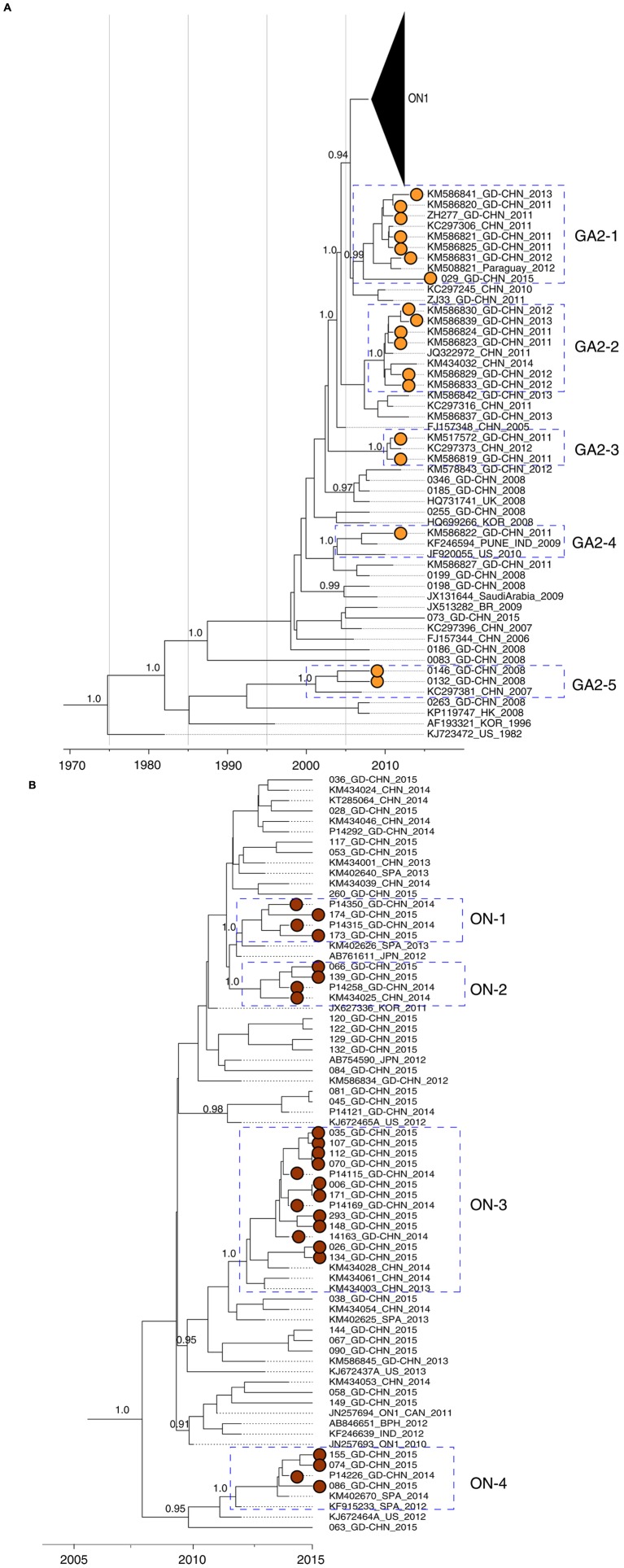
**Molecular clock phylogeny of *G* gene sequences from Guangdong between 2008 and 2015**. Phylogeny was estimated using maximum Clade Credibility (MCC) on the basis of *G* gene sequences obtained from Guangdong between 2008 and 2015 and closely related sequences from other regions. Posterior values of interested clusters are shown next to nodes. Guangdong RSV-A strains in interested clusters are highlighted with dots filled with colors. **(A)** Viral strains belong to the new emerged ON1 genotype is shown as a triangle. The interested clusters of RSV-A strains with high posterior values are highlighted with boxes and denoted. **(B)** Phylogeny of ON1 genotype RSV-A viruses. The interested clusters are highlighted and denoted.

The above analyses provided a basic overview on RSV-A evolution and transmission on a global scale. To further understand how RSV-A viruses circulated in a local area, we also estimated MCC phylogenetic trees from RSV-A sequences from Guangdong, China, together with closely related sequences from other regions. All Guangdong strains collected in 2008–2015 belong to genotype GA2. However, these strains were segregated into multiple subclusters and strains collected from the same epidemic season fell into different subclusters (**Figure 3A**). For example, strain 0263_GD-CHN_2008 is phylogenetically quite distinct from other strains collected in Guangdong 2008 such as 0185 and 0198, which grouped with contemporary strains from other countries.

In the phylogenetic tree (**Figure 3A**), we found several local clusters (GA2-2, GA2-3, and GA2-5) of Guangdong sequences, and also clusters that contained both Guangdong and non-Guangdong strains (GA2-1 and GA2-4). Local clusters contained viral strains collected from successive epidemic seasons, e.g., from 2011 to 2015 in the GA2-1 cluster. One interpretation of this result is that GA2 viruses have persisted in Guangdong between seasons. However, it is also equally possible that each local cluster was imported into Guangdong multiple times, but these importations are not observed due to the limited sampling of RSV from other locations. The presence of a sequence from Paraguay in cluster GA2-1 support the idea that there is substantial international movement of RSV lineages that is not being detected in current data due to limited virus sequence data from many regions. In the GA2-4 cluster, strain KM586822_GD-CHN_2011 is closely related to external strains sampled in the USA and India collected in previous years.

**FIGURE 4:**
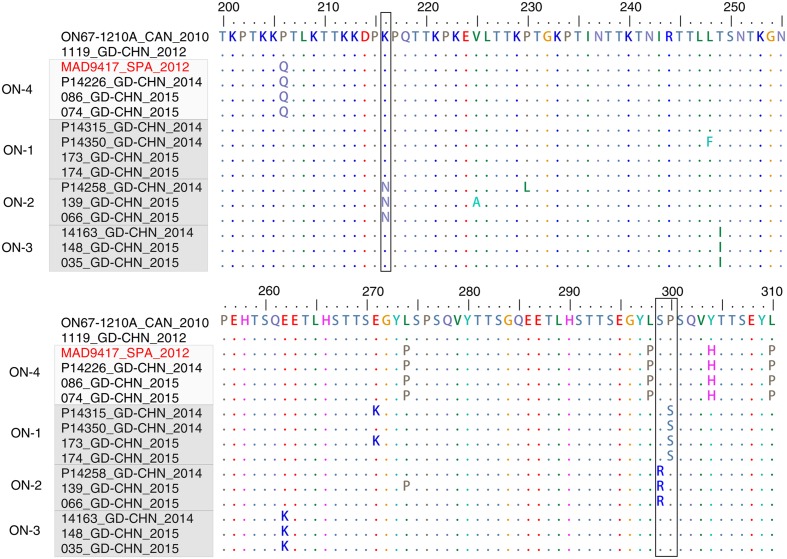
**G-protein sequences alignment of ON1 genotype viruses**. Representative Guangdong ON1 RSV-A strains and closely related strains are included. The highly variable region in the C-terminal of G-protein sequences (200–310, corresponding to the strain ON67-1210A_2010, accession number JN257693) is shown. The viruses are clustered according to the result of **Figure 3B**. The positions which are exclusively found in Guangdong strains are highlighted in black box.

A similar pattern was observed for ON1 subgenotype viruses detected in Guangdong. The ON1 genotype is characterized by a 72 nt insertion in the viral *G* gene, resulting in 24 additional amino acids, of which 23 are duplications of amino acid positions 261–283 (Eshaghi et al., 2012). This new variant was first detected in Guangdong in 2012 (**Figure 3B**), and was predominant in Guangdong samples between 2014 and 2015, accounting for 9 of 9 strains sequenced in 2014 and for 36 of 38 (95%) strains analyzed in 2015 (**Figure 3B**). Local clusters of Guangdong strains collected between 2014 and 2015 were observed in the ON1 genotype (ON-1, ON-2, and ON-3, **Figure 3B**). In addition, Guangdong strains related to P14226_GD-CHN_2014 were more closely to a strain identified in Spain in 2012 (KF915233_SPA_2012) and this cluster is correspondingly termed ON-4. As discussed above, the amount of regional and international mixing of RSV observed in the phylogeny is likely underestimated due to limited sampling in many regions, although the highly similar sequences in cluster ON-3 probably do represent circulation within Guangdong or China itself, during the epidemics in 2014 and 2015. The amino acid alignment of the G protein of ON1 strains matches the corresponding phylogeny. **Figure 4** shows part of an alignment containing the variable mucin-like domains of the G protein (McLellan et al., 2013). The earliest ON1 strain from Guangdong (1119_GD-CHN_2012) shows 100% amino acid identity (across the full length of the G protein) to the first ON1 strain, identified in Canada in 2010. Viral strains within the clusters defined in **Figure 3B** show the same or highly similar amino acid changes. Several substitutions are specific to Guangdong and Chinese RSV strains belong to the ON1 genotype, specifically Lys216Asn, Ser299Arg and Pro300Ser.

## Discussion

RSV is one of the most important respiratory pathogen worldwide. Compared to human influenza viruses, the molecular epidemiology of RSV is largely unknown at both local and global scales. In this study, we undertook phylogenetic, spatial and molecular clock analyses on RSV-A by using sequences data from public database and from the surveillance in Guangdong China between 2008 and 2015. To achieve robust results in molecular clock and phylogeographic analyses, we used complete or nearly complete (>95%) *G* gene sequences in this study. RSV-A strains with partial sequences or without information on the date and location of sampling were not included. As a consequence, some genotypes like GA3, GA4, and GA6, which may include one or a few sequences, were not represented in the phylogenetic tree. The importance of these sequences is limited because these genotypes (present as minor clades in phylogenies of partial *G* gene sequences) were rarely detected after 2010 in epidemiological studies (Trento et al., 2015).

At the global scale, different circulation patterns are observed for different genotypes of RSV-A. Genotypes such as GA1, GA4, and GA5, are more endemic and display limited epidemic activity throughout their history (**Figure 2**). All appear to have originated and mainly circulated in America, with a few sequences identified on other continents, although this conclusion will not be reliable if early molecular surveillance of RSV was strongly biased toward infections in the USA. However, a distinct pattern is observed for the GA2 genotype, which appears to be more geographically widespread and epidemically active (Eshaghi et al., 2012; Houspie et al., 2013; Trento et al., 2015). Molecular clock analysis reveals that circulation of RSV-A can be classified into three distinct periods (**Figure 2**). Before 1990, RSV-A most sequenced infection cases were mainly identified in America. Between 1990 and 2005, driven by the emergence of GA2, RSV-A viruses were increasingly detected in countries on different continents and temporary co-circulation of different genotypes is observed. The most significant feature of RSV-A molecular epidemiology is the predominance of GA2 genotype after 2005 (Eshaghi et al., 2012; Houspie et al., 2013; Agoti et al., 2014; Liu et al., 2014; Pierangeli et al., 2014; Duvvuri et al., 2015). The prevalence of the GA2 genotype in a population may inhibit the circulation of other genotypes, such as GA7 and GA5, which now are rarely detected, even in America. One possible explanation for this could be the immune cross-protection in a in population generated by GA2 infection. A recent *in vitro* study by Treno et al. (2015) suggested that an antibody (MON-3-88) generated by GA2 virus infection exhibited a broad reactivity to other genotypes including GA3, GA5, and GA7, but not to GA1.

Our study benefits from the continuous surveillance of RSV in Guangdong, enabling us to investigate the genetic diversity of RSV-A across different epidemic seasons in a defined region. Our results indicate that local RSV-A epidemics are caused by a combination of local virus persistence and repeated re-introductions from external locations.

As studies of influenza A viruses have shown (Nelson et al., 2006, 2007; Russell et al., 2008), a simple test of persistence versus seeding is to examine the phylogenetic relationships of strains sampled between epidemics. If epidemics are mainly caused by virus persistence, new emerged strains would be descended from, and thus more closely related to, strains from previous epidemic in this area. Conversely, if epidemic strains are related to contemporary strains from outside, the epidemics are more likely caused by virus importations.

The phylogenetic analysis highlights both local and global clusters are identified during RSV-A epidemics in Guangdong (**Figures 3A,B**). In addition, the phylogenetic tree mainly based on the Guangdong RSV-A strains (**Figure 2**) is quite similar with the full phylogeny tree of GA2 genotype at global level (**Figure 3**; Supplementary Figure S1). Some Guangdong strains collected in 2008 are near the root of GA2 genotype while the recent sequences collected between 2011 and 2015 in Guangdong fall into the major contemporary cluster of GA2 genotype. One interpretation of this pattern is that the RSV-A circulation in Guangdong is in equilibrium with global RSV-A virus distribution. An alternative but more complex explanation is that viral strains in Guangdong are under parallel evolution after the early dissemination occurred before 2008. The molecular epidemiology of new emerged ON1 suggests the former explanation should be preferred. The ON1 genotype was first detected in Ontario in the winter of 2010/11 (Eshaghi et al., 2012). Thereafter the genotype has been widely spread and prevalent in South Africa (Agoti et al., 2014), Germany (Prifert et al., 2013), Italy (Pierangeli et al., 2014), and Malaysia (Khor et al., 2013). The ON1 RSV-A virus was detected in Guangdong in 2012 and possesses the G protein with the 100% amino acid sequence identity of the Canadian strain suggesting that the novel sub-genotype ON1 virus detected in Guangdong in 2012 is more likely from external seeding. After the possible early introductions, some ON1 strains collected in Guangdong in 2014 and 2015 possess a few amino acid substitutions in G protein are exclusively found in Guangdong and Beijing city (Cui et al., 2015) of China between 2014 and 2015. The prevalence of ON1 genotype in Guangdong also led to the increasing of RSV clinical infection cases and positive rate of RSV in ARIs, 2014–2015. Interestingly, the most recent study from Kenya also suggested both new introductions and local persistence of RSV-A viruses contribute the recurrent of epidemics (Otieno et al., 2016). However, it should be also noted that current phylogenies likely substantially underestimate the contribution of re-introductions to a given location (e.g., Guangdong) due to low levels of sequence sampling from many countries and regions. More representative sampling across the globe, or within a more geographically confined area of interest, will provide more robust results on transmission pattern of RSV-A virus. Another limitation should be emphasized is that our analysis do not have extensive Guangdong RSV-A sequences even though large number of ARIs samples are collected (Supplementary Table S1). This is a common problem for RSV epidemiology studies, partly because a wide range of pathogens can cause ARIs, including bacteria and other viruses besides RSV such as Influenza viruses, parainfluenza virus, adenovirus and human rhinoviruses (van den Hoogen et al., 2001; Azziz-Baumgartner et al., 2012; Kwofie et al., 2012; Marcone et al., 2012). However, the limit surveillance data in Guangdong has already shown some local clusters of RSV with several distinct mutations (**Figures 3** and **4**) and the ON1 prevalence in Guangdong following its emergence in Canada. These results provide the evidence of the joint transmission pattern of RSV-A in Guangdong.

Currently, the RSV GA2 genotype that circulate worldwide has turned into a predominant RSV-A genotype with new variants emerging through a process of continuous evolution that appears somewhat similar to that observed for the global circulation of seasonal influenza viruses (Russell et al., 2008) and human noroviruses (Siebenga et al., 2010; Lu et al., 2016). Our local data suggest RSV-A epidemics in Guangdong are caused by viruses seeded from external regions and viruses persist in local jointly. In this context, for global RSV disease control and vaccine development, much more detailed surveys of RSV-A genetic diversity and evolution in all affected areas, comparable to the surveillance undertaken here for Guangdong province, should be encouraged.

## Author Contributions

LZ and CK designed the study. LZ, LY, JW, YS, GH, XZ, LL, and HN prepared sample collection and genome sequencing. LY and JL analyzed the data. JL and OP interpreted the data. JL and OP wrote the paper. All authors reviewed the manuscript.

## Conflict of Interest Statement

The authors declare that the research was conducted in the absence of any commercial or financial relationships that could be construed as a potential conflict of interest.

## Footnotes

1 http://www.ncbi.nlm.nih.gov/genbank

2 http://www.datamonkey.org/

3 tree.bio.ed.ac.uk
