# Supplementary material for: Evolution and Transmission of Respiratory Syncytial Group A (RSV-A) Viruses in Guangdong, China 2008–2015
Source: Front Microbiol. 2016 Aug 15;7:1263. doi: 10.3389/fmicb.2016.01263 (PMC4983572; doi:10.3389/fmicb.2016.01263)
Supplement: Supplementary file 1 [file Table_1.DOCX]

Table S1. Number of respiratory samples collected from ARIs suspicious patients each year and relative positive rate for RSV infection.

| **Year** | **ARIs Patients No.** | **RSV Positive****, No. (%)** |
| --- | --- | --- |
|  | (n = 3843) | (n = 295) |
| 2008 | 503 | 43 (8.5) |
| 2009 | 425 | 14 (3.3) |
| 2010 | 393 | 9 (2.3) |
| 2011 | 442 | 27 (6.1) |
| 2012 | 497 | 21 (4.2) |
| 2013 | 477 | 26 (5.5) |
| 2014 | 622 | 88 (14.1) |
| 2015 | 484 | 67 (13.8) |

Table S2. Characteristics of RSV infection patients in Guangdong, 2008-2015.

| **Characteristics** | **Patients** |
| --- | --- |
|  | n = 295 (%) |
| **Sex** |  |
| Male | 171 (58.0） |
| Female | 124 (42.0) |
| **Age group** |  |
| 0–4 years | 117 (39.7) |
| 5–14 years | 43 (14.6) |
| 15–24 years | 29 (9.8) |
| 25–49 years | 59 (20.0) |
| 50–64 years | 23 (7.8) |
| ≥70 years | 24 (8.1) |

Table S3. Accession number of G gene sequences used in the phylogenetic analysis in this study.

| \| AB754590 \| HQ699275 \| JX198110 \| KC297386 \| KF246612 \| KF915221 \| KJ627690 \| KM042392 \| KM434060 \| KP258740 \| \| --- \| --- \| --- \| --- \| --- \| --- \| --- \| --- \| --- \| --- \| \| AB761609 \| HQ699276 \| JX198112 \| KC297394 \| KF246613 \| KF915231 \| KJ627691 \| KM360090 \| KM434061 \| KP258743 \| \| AB761610 \| HQ699279 \| JX198114 \| KC297395 \| KF246615 \| KF915233 \| KJ627693 \| KM402620 \| KM508816 \| KP258744 \| \| AB761611 \| HQ699280 \| JX198118 \| KC297396 \| KF246618 \| KF915234 \| KJ627699 \| KM402621 \| KM508817 \| KP317933 \| \| AB846651 \| HQ699284 \| JX198119 \| KC297400 \| KF246638 \| KF915235 \| KJ627701 \| KM402623 \| KM508821 \| KP317936 \| \| AB846652 \| HQ699285 \| JX198126 \| KC297402 \| KF246639 \| KF915236 \| KJ627707 \| KM402625 \| KM508824 \| KP317944 \| \| AB846656 \| HQ699286 \| JX198129 \| KC297404 \| KF246640 \| KF915247 \| KJ627709 \| KM402626 \| KM508825 \| KP317948 \| \| AB846657 \| JF714706 \| JX198134 \| KC297411 \| KF246642 \| KF915249 \| KJ627710 \| KM402627 \| KM517572 \| KP317949 \| \| AB846662 \| JF920046 \| JX198135 \| KC297418 \| KF300972 \| KF915251 \| KJ627713 \| KM402630 \| KM578843 \| KP317950 \| \| AF065405 \| JF920049 \| JX198136 \| KC297420 \| KF530260 \| KF915259 \| KJ627723 \| KM402633 \| KM586819 \| KP317951 \| \| AF065406 \| JF920053 \| JX198137 \| KC297421 \| KF530261 \| KF973332 \| KJ627730 \| KM402636 \| KM586820 \| KP663728 \| \| AF065407 \| JF920055 \| JX513282 \| KC476659 \| KF530268 \| KF973334 \| KJ627731 \| KM402637 \| KM586821 \| KP792352 \| \| AF065409 \| JF920057 \| JX627336 \| KC476661 \| KF826817 \| KJ627257 \| KJ627732 \| KM402639 \| KM586822 \| KP792355 \| \| AF065410 \| JF920059 \| JX976338 \| KC476665 \| KF826821 \| KJ627259 \| KJ627736 \| KM402640 \| KM586823 \| KP792356 \| \| AF193305 \| JF920061 \| JX976341 \| KC476670 \| KF826823 \| KJ627260 \| KJ627737 \| KM402641 \| KM586824 \| KP792357 \| \| AF193307 \| JF920065 \| JX976343 \| KC476684 \| KF826826 \| KJ627264 \| KJ643588 \| KM402644 \| KM586825 \| KP792359 \| \| AF193308 \| JF920067 \| JX976344 \| KC476689 \| KF826828 \| KJ627270 \| KJ672428 \| KM402651 \| KM586827 \| KP792360 \| \| AF193310 \| JF979145 \| JX976346 \| KC476691 \| KF826832 \| KJ627271 \| KJ672429 \| KM402655 \| KM586828 \| KP792362 \| \| AF193311 \| JF979148 \| JX976349 \| KC476697 \| KF826833 \| KJ627272 \| KJ672433 \| KM402663 \| KM586829 \| KP792363 \| \| AF193313 \| JF979151 \| JX976350 \| KC476728 \| KF826837 \| KJ627274 \| KJ672434 \| KM402666 \| KM586830 \| KP792365 \| \| AF193315 \| JF979152 \| JX976360 \| KC476731 \| KF826838 \| KJ627276 \| KJ672435 \| KM402667 \| KM586831 \| KP792367 \| \| AF193316 \| JF979155 \| JX976363 \| KC476733 \| KF826840 \| KJ627287 \| KJ672436 \| KM402670 \| KM586832 \| KP792370 \| \| AF193318 \| JF979156 \| JX976364 \| KC476745 \| KF826841 \| KJ627288 \| KJ672437 \| KM434000 \| KM586833 \| KP792372 \| \| AF193319 \| JF979157 \| KC283043 \| KC559440 \| KF826847 \| KJ627291 \| KJ672440 \| KM434001 \| KM586834 \| KP792375 \| \| AF193320 \| JN257696 \| KC297233 \| KC559442 \| KF826848 \| KJ627293 \| KJ672448 \| KM434002 \| KM586837 \| KP792376 \| \| AF193321 \| JN257697 \| KC297234 \| KC559445 \| KF826856 \| KJ627294 \| KJ672458 \| KM434003 \| KM586839 \| KP856967 \| \| AF193324 \| JN257702 \| KC297238 \| KC559447 \| KF915152 \| KJ627304 \| KJ672464 \| KM434006 \| KM586841 \| KP856968 \| \| AF193326 \| JQ322971 \| KC297245 \| KC559448 \| KF915153 \| KJ627308 \| KJ672465 \| KM434007 \| KM586842 \| KP856969 \| \| AY344657 \| JQ322972 \| KC297260 \| KC677854 \| KF915156 \| KJ627315 \| KJ672469 \| KM434008 \| KM586845 \| KT285064 \| \| AY344658 \| JQ901447 \| KC297267 \| KC677855 \| KF915157 \| KJ627316 \| KJ672471 \| KM434009 \| KP119745 \| M17212 \| \| AY344662 \| JQ901448 \| KC297288 \| KC677863 \| KF915159 \| KJ627320 \| KJ672472 \| KM434010 \| KP119746 \| U39662 \| \| AY344663 \| JQ901449 \| KC297292 \| KC677865 \| KF915163 \| KJ627327 \| KJ672479 \| KM434013 \| KP119747 \| X73351 \| \| AY571777 \| JQ901451 \| KC297304 \| KC677866 \| KF915165 \| KJ627335 \| KJ723461 \| KM434020 \| KP119748 \| X73352 \| \| EF650648 \| JQ901452 \| KC297306 \| KC677867 \| KF915166 \| KJ627336 \| KJ723462 \| KM434024 \| KP164503 \| X73353 \| \| FJ157344 \| JQ901454 \| KC297311 \| KC677870 \| KF915167 \| KJ627344 \| KJ723464 \| KM434025 \| KP164504 \| X73354 \| \| FJ157345 \| JQ901455 \| KC297316 \| KC677874 \| KF915171 \| KJ627350 \| KJ723472 \| KM434028 \| KP164505 \| X73355 \| \| FJ157348 \| JQ901456 \| KC297317 \| KC677877 \| KF915173 \| KJ627351 \| KJ723474 \| KM434032 \| KP164507 \| Z33412 \| \| GU591759 \| JQ901457 \| KC297333 \| KC731482 \| KF915174 \| KJ627353 \| KJ723478 \| KM434033 \| KP218910 \| Z33415 \| \| GU591760 \| JQ901458 \| KC297334 \| KC731483 \| KF915177 \| KJ627355 \| KJ723486 \| KM434035 \| KP258696 \| Z33417 \| \| GU591763 \| JX015481 \| KC297335 \| KC754738 \| KF915181 \| KJ627358 \| KJ939943 \| KM434039 \| KP258699 \| Z33418 \| \| GU591766 \| JX015487 \| KC297337 \| KC754739 \| KF915184 \| KJ627360 \| KJ939949 \| KM434042 \| KP258700 \| Z33419 \| \| GU591768 \| JX015492 \| KC297347 \| KC754741 \| KF915186 \| KJ627361 \| KJ939955 \| KM434045 \| KP258704 \| Z33420 \| \| GU591769 \| JX069799 \| KC297348 \| KC754743 \| KF915187 \| KJ627366 \| KJ939958 \| KM434046 \| KP258707 \| Z33421 \| \| GU591770 \| JX069802 \| KC297354 \| KC763335 \| KF915190 \| KJ627367 \| KJ939961 \| KM434048 \| KP258709 \| Z33422 \| \| GU591771 \| JX131638 \| KC297355 \| KC763339 \| KF915194 \| KJ627369 \| KJ939963 \| KM434049 \| KP258710 \| Z33423 \| \| HQ699265 \| JX131640 \| KC297361 \| KC978856 \| KF915197 \| KJ627374 \| KJ939966 \| KM434052 \| KP258723 \| Z33425 \| \| HQ699266 \| JX131641 \| KC297366 \| KF246587 \| KF915203 \| KJ627647 \| KJ939969 \| KM434053 \| KP258726 \| Z33428 \| \| HQ699270 \| JX131644 \| KC297373 \| KF246594 \| KF915213 \| KJ627656 \| KM042381 \| KM434054 \| KP258727 \| Z33429 \| \| HQ699271 \| JX198105 \| KC297382 \| KF246604 \| KF915214 \| KJ627668 \| KM042382 \| KM434055 \| KP258729 \| Z33432 \| \| HQ699273 \| JX198108 \| KC297384 \| KF246605 \| KF915215 \| KJ627682 \| KM042386 \| KM434058 \| KP258733 \| \| \| HQ699274 \| JX198109 \| KC297385 \| KF246606 \| KF915220 \| KJ627685 \| KM042389 \| KM434059 \| KP258737 \| \| |
| --- | --- | --- | --- | --- | --- | --- | --- | --- | --- | --- | --- | --- | --- | --- | --- | --- | --- | --- | --- | --- | --- | --- | --- | --- | --- | --- | --- | --- | --- | --- | --- | --- | --- | --- | --- | --- | --- | --- | --- | --- | --- | --- | --- | --- | --- | --- | --- | --- | --- | --- | --- | --- | --- | --- | --- | --- | --- | --- | --- | --- | --- | --- | --- | --- | --- | --- | --- | --- | --- | --- | --- | --- | --- | --- | --- | --- | --- | --- | --- | --- | --- | --- | --- | --- | --- | --- | --- | --- | --- | --- | --- | --- | --- | --- | --- | --- | --- | --- | --- | --- | --- | --- | --- | --- | --- | --- | --- | --- | --- | --- | --- | --- | --- | --- | --- | --- | --- | --- | --- | --- | --- | --- | --- | --- | --- | --- | --- | --- | --- | --- | --- | --- | --- | --- | --- | --- | --- | --- | --- | --- | --- | --- | --- | --- | --- | --- | --- | --- | --- | --- | --- | --- | --- | --- | --- | --- | --- | --- | --- | --- | --- | --- | --- | --- | --- | --- | --- | --- | --- | --- | --- | --- | --- | --- | --- | --- | --- | --- | --- | --- | --- | --- | --- | --- | --- | --- | --- | --- | --- | --- | --- | --- | --- | --- | --- | --- | --- | --- | --- | --- | --- | --- | --- | --- | --- | --- | --- | --- | --- | --- | --- | --- | --- | --- | --- | --- | --- | --- | --- | --- | --- | --- | --- | --- | --- | --- | --- | --- | --- | --- | --- | --- | --- | --- | --- | --- | --- | --- | --- | --- | --- | --- | --- | --- | --- | --- | --- | --- | --- | --- | --- | --- | --- | --- | --- | --- | --- | --- | --- | --- | --- | --- | --- | --- | --- | --- | --- | --- | --- | --- | --- | --- | --- | --- | --- | --- | --- | --- | --- | --- | --- | --- | --- | --- | --- | --- | --- | --- | --- | --- | --- | --- | --- | --- | --- | --- | --- | --- | --- | --- | --- | --- | --- | --- | --- | --- | --- | --- | --- | --- | --- | --- | --- | --- | --- | --- | --- | --- | --- | --- | --- | --- | --- | --- | --- | --- | --- | --- | --- | --- | --- | --- | --- | --- | --- | --- | --- | --- | --- | --- | --- | --- | --- | --- | --- | --- | --- | --- | --- | --- | --- | --- | --- | --- | --- | --- | --- | --- | --- | --- | --- | --- | --- | --- | --- | --- | --- | --- | --- | --- | --- | --- | --- | --- | --- | --- | --- | --- | --- | --- | --- | --- | --- | --- | --- | --- | --- | --- | --- | --- | --- | --- | --- | --- | --- | --- | --- | --- | --- | --- | --- | --- | --- | --- | --- | --- | --- | --- | --- | --- | --- | --- | --- | --- | --- | --- | --- | --- | --- | --- | --- | --- | --- | --- | --- | --- | --- | --- | --- | --- | --- | --- | --- | --- | --- | --- | --- | --- | --- | --- | --- | --- | --- | --- | --- | --- | --- | --- | --- | --- | --- | --- | --- | --- | --- | --- | --- | --- | --- | --- | --- | --- | --- | --- | --- | --- | --- | --- | --- | --- | --- | --- | --- | --- | --- | --- | --- | --- | --- | --- | --- | --- | --- | --- | --- | --- | --- | --- | --- | --- | --- | --- | --- | --- | --- | --- | --- | --- | --- | --- | --- | --- | --- | --- | --- | --- | --- | --- | --- | --- |
