# Supplementary material for: Evolution and Transmission of Respiratory Syncytial Group A (RSV-A) Viruses in Guangdong, China 2008–2015
Source: Front Microbiol. 2016 Aug 15;7:1263. doi: 10.3389/fmicb.2016.01263 (PMC4983572; doi:10.3389/fmicb.2016.01263)
Supplement: Supplementary file 2 [file Image_1.PDF]

Figure S1. **Molecular clock phylogeny of *G* gene sequences from Guangdong between 2008 and 2015.** Phylogeny was estimated using maximum Clade Credibility (MCC) on the basis of *G* gene sequences obtained from Guangdong between 2008 and 2015 and closely related sequences from other regions. The GA2 and ON1 genotypes were noted.

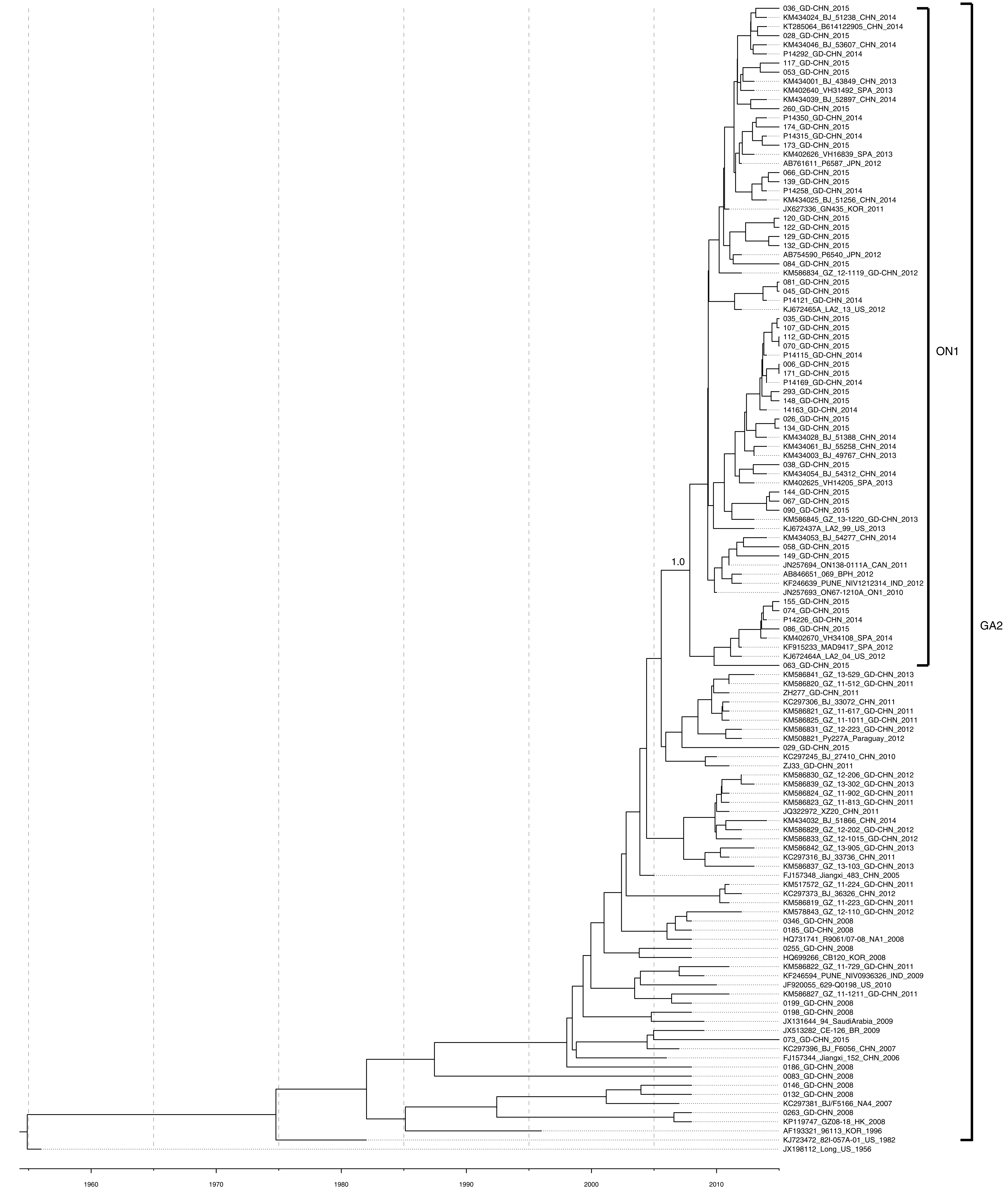

036_GD-CHN_2015
KM434024_BJ_51238_CHN_2014
KT285064_B614122905_CHN_2014
028_GD-CHN_2015
KM434046_BJ_53607_CHN_2014
P14292_GD-CHN_2014
117_GD-CHN_2015
053_GD-CHN_2015
KM434001_BJ_43849_CHN_2013
KM402640_VH31492_SPA_2013
KM434039_BJ_52897_CHN_2014
260_GD-CHN_2015
P14350_GD-CHN_2014
174_GD-CHN_2015
P14315_GD-CHN_2014
173_GD-CHN_2015
KM402626_VH16839_SPA_2013
AB761611_P6587_JPN_2012
066_GD-CHN_2015
139_GD-CHN_2015
P14258_GD-CHN_2014
KM434025_BJ_51256_CHN_2014
JX627336_GN435_KOR_2011
120_GD-CHN_2015
122_GD-CHN_2015
129_GD-CHN_2015
132_GD-CHN_2015
AB754590_P6540_JPN_2012
084_GD-CHN_2015
KM586834_GZ_12-1119_GD-CHN_2012
081_GD-CHN_2015
045_GD-CHN_2015
P14121_GD-CHN_2014
KJ672465A_LA2_13_US_2012
035_GD-CHN_2015
107_GD-CHN_2015
112_GD-CHN_2015
070_GD-CHN_2015
P14115_GD-CHN_2014
006_GD-CHN_2015
171_GD-CHN_2015
P14169_GD-CHN_2014
293_GD-CHN_2015
148_GD-CHN_2015
14163_GD-CHN_2014
026_GD-CHN_2015
134_GD-CHN_2015
KM434028_BJ_51388_CHN_2014
KM434061_BJ_55258_CHN_2014
KM434003_BJ_49767_CHN_2013
038_GD-CHN_2015
KM434054_BJ_54312_CHN_2014
KM402625_VH14205_SPA_2013
144_GD-CHN_2015
067_GD-CHN_2015
090_GD-CHN_2015
KM586845_GZ_13-1220_GD-CHN_2013
KJ672437A_LA2_99_US_2013
KM434053_BJ_54277_CHN_2014
058_GD-CHN_2015
149_GD-CHN_2015
JN257694_ON138-0111A_CAN_2011
AB846651_069_BPH_2012
KF246639_PUNE_NIV1212314_IND_2012
JN257693_ON67-1210A_ON1_2010
155_GD-CHN_2015
074_GD-CHN_2015
P14226_GD-CHN_2014
086_GD-CHN_2015
KM402670_VH34108_SPA_2014
KF915233_MAD9417_SPA_2012
KJ672464A_LA2_04_US_2012
063_GD-CHN_2015
KM586841_GZ_13-529_GD-CHN_2013
KM586820_GZ_11-512_GD-CHN_2011
ZH277_GD-CHN_2011
KC297306_BJ_33072_CHN_2011
KM586821_GZ_11-617_GD-CHN_2011
KM586825_GZ_11-1011_GD-CHN_2011
KM586831_GZ_12-223_GD-CHN_2012
KM508821_Py227A_Paraguay_2012
029_GD-CHN_2015
KC297245_BJ_27410_CHN_2010
ZJ33_GD-CHN_2011
KM586830_GZ_12-206_GD-CHN_2012
KM586839_GZ_13-302_GD-CHN_2013
KM586824_GZ_11-902_GD-CHN_2011
KM586823_GZ_11-813_GD-CHN_2011
JQ322972_XZ20_CHN_2011
KM434032_BJ_51866_CHN_2014
KM586829_GZ_12-202_GD-CHN_2012
KM586833_GZ_12-1015_GD-CHN_2012
KM586842_GZ_13-905_GD-CHN_2013
KC297316_BJ_33736_CHN_2011
KM586837_GZ_13-103_GD-CHN_2013
FJ157348_Jiangxi_483_CHN_2005
KM517572_GZ_11-224_GD-CHN_2011
KC297373_BJ_36326_CHN_2012
KM586819_GZ_11-223_GD-CHN_2011
KM578843_GZ_12-110_GD-CHN_2012
0346_GD-CHN_2008
0185_GD-CHN_2008
HQ731741_R9061/07-08_NA1_2008
0255_GD-CHN_2008
HQ699266_CB120_KOR_2008
KM586822_GZ_11-729_GD-CHN_2011
KF246594_PUNE_NIV0936326_IND_2009
JF920055_629-Q0198_US_2010
KM586827_GZ_11-1211_GD-CHN_2011
0199_GD-CHN_2008
0198_GD-CHN_2008
JX131644_94_SaudiArabia_2009
JX513282_CE-126_BR_2009
073_GD-CHN_2015
KC297396_BJ_F6056_CHN_2007
FJ157344_Jiangxi_152_CHN_2006
0186_GD-CHN_2008
0083_GD-CHN_2008
0146_GD-CHN_2008
0132_GD-CHN_2008
KC297381_BJ/F5166_NA4_2007
0263_GD-CHN_2008
KP119747_GZ08-18_HK_2008
AF193321_96113_KOR_1996
KJ723472_82I-057A-01_US_1982
JX198112_Long_US_1956
1.0
ON1
GA2
1960
1970
1980
1990
2000
2010
